# Bacterial enzyme-responsive hydrogels for triggered delivery of antibiotics to infected wounds

**DOI:** 10.1126/sciadv.adz0786

**Published:** 2026-03-20

**Authors:** Akram Abbasi, Alec McCall, Zhaowei Jiang, Brian W. LeBlanc, Anita Shukla

**Affiliations:** ^1^ School of Engineering, Institute for Biology, Engineering, and Medicine, Brown University, Providence, RI, USA.; ^2^ Department of Surgery, Division of Surgical Research, Rhode Island hospital, Providence, RI, USA.

## Abstract

Wound infections are becoming increasingly difficult to treat due to rising antibiotic-resistant bacteria. β-Lactamase–producing bacteria are among the most common pathogens implicated in these infections. Here, we report a bacterial enzyme-responsive hydrogel formulated with a cephalosporin-derived, β-lactamase–cleavable crosslinker that undergoes selective degradation in the presence of bacterial β-lactamases. This degradation triggers the on-demand release of encapsulated ciprofloxacin-loaded liposomes, ensuring that antibiotic delivery occurs only at the site of infection. This selective degradation and release was demonstrated in both ex vivo and in vivo models of *Pseudomonas aeruginosa* wound infections. In a murine skin abrasion infection model, a single application of the hydrogel led to complete bacterial eradication and enhanced wound healing, outperforming a commercial silver-based hydrogel wound dressing. These responsive hydrogels did not induce ciprofloxacin resistance in non–β-lactamase–producing bacteria. These findings demonstrate that β-lactamase–responsive hydrogels provide a precise, infection-triggered antibiotic delivery platform that can improve the treatment of wound infections and mitigate antimicrobial resistance.

## Teaser

Hydrogel delivers antibiotics on-demand to infected wounds in response to bacterial enzymes mitigating antimicrobial resistance.

## INTRODUCTION

Rising antibiotic-resistant infections are a critical public health challenge. If no action is taken, it is projected that these infections could result in 10 million deaths annually by 2050 (*1*). This alarming trend is largely attributed to the overuse of antibiotics in various settings, including wound management (*2*). Alarmingly, ~50% of wound infections are now resistant to potent antimicrobials, such as third-generation cephalosporins, underscoring the growing concern of antibiotic resistance in wound infections (*3*). Despite this startling statistic, β-lactam antibiotics remain the most widely prescribed class of antibiotics used in treating wound and skin/soft tissue infections, including penicillins (e.g., amoxicillin), cephalosporins (e.g., cefazolin and ceftazidime), carbapenems (e.g., imipenem and meropenem), and monobactams (e.g., aztreonam) (*4*, *5*). However, their clinical utility is increasingly compromised by growing β-lactam resistance in common wound pathogens (*6*–*8*).

β-Lactamase enzyme production is one of the primary bacterial resistance mechanisms against β-lactam antibiotics (*4*, *5*). These antibiotics feature a β-lactam ring in their chemical structure, which mimics the substrate of the bacterial penicillin-binding protein, interfering with bacterial cell wall crosslinking. β-Lactamases hydrolyze the amide bond in the β-lactam ring, rendering β-lactam antibiotics ineffective (*8*). Bacteria mutations and increased β-lactamase gene expression under selective antibiotic pressure, combined with the dissemination of β-lactamase genes through horizontal gene transfer, have led to the widespread prevalence of β-lactamases in various bacterial species, including most of the most common wound infection causing bacteria, including *Pseudomonas aeruginosa*, *Klebsiella pneumoniae*, and methicillin-resistant *Staphylococcus aureus* (*6*–*8*). This rising prevalence of β-lactamase–mediated resistance underscores the need for alternative therapeutic strategies that enable effective treatment of infections caused by β-lactamase–producing bacteria while limiting further resistance development.

Hydrogels have emerged as promising candidates for antimicrobial wound dressings that enable local wound treatment (*9*). These three-dimensional (3D) water swollen networks of hydrophilic polymers offer a moist environment for wound healing, high oxygen permeability, and the potential to encapsulate and deliver antimicrobial agents directly at the wound site. This localized delivery minimizes systemic antimicrobial exposure, which can be toxic and disrupt the microbiota (*10*). However, current clinically used antimicrobial hydrogel dressings predominantly operate via a passive release mechanism, resulting in a lack of precise control over drug delivery and diminished specificity, leading to suboptimal therapeutic outcomes and contributing to the spread of antibiotic resistant superbugs (*11*). Stimuli-responsive hydrogels, which interact with the dynamic environment of the wound site, have the potential to overcome these challenges, offering a notable advance in wound care, particularly for infection management (*12*). This “smart” approach to drug delivery ensures that medications are deployed precisely when and where they are needed, enhancing efficacy and minimizing side effects.

“Smart” hydrogels can be engineered to recognize specific signals within the wound milieu, such as pH levels and the presence of reactive oxygen species (ROS) (*13*–*16*) and microbial enzymes (*17*–*19*), to initiate the release of therapeutic agents. Extensive research has been conducted on hydrogels designed for pH-triggered drug release, often using acid-labile bonds such as Schiff base linkages or leveraging principles of supramolecular chemistry to release drugs in the acidic microenvironment of most infected wounds (*20*, *21*). However, note that fluctuations in pH or ROS levels can also occur in noninfected healing wounds, bringing into question the specificity of these triggers for drug release in infected wounds (*22*). In response, bacterial enzymes have emerged as a highly selective trigger for controlled drug release (*23*). For example, Xiong *et al.* (*24*) developed nanogels with a polyphosphoester crosslinked core that undergo hydrolysis upon interaction with bacterial lipases and phospholipases, leading to the release of antibacterial agents. Recently, Huang *et al.* (*25*) developed a protease-responsive protein hydrogel to respond to local protease activity in inflammatory microenvironments.

Many of the enzymes explored as potential triggers for antimicrobial delivery from hydrogels share functional similarities with mammalian counterparts, including proteases involved in wound healing (*26*). To provide a truly precise bacteria-triggered drug delivery mechanism, which can reduce unnecessary drug exposure to healthy mammalian tissue and better target pathogenic bacteria, it is critical to select enzyme triggers that discriminate between bacterial and mammalian enzymatic activity. β-Lactamases are an attractive option, as an enzyme commonly produced by the most prevalent wound infection causing bacteria and a leading cause of antibiotic resistance. β-Lactam hydrolysis has only recently been explored as a mechanism of stimuli responsiveness. The hydrolytic cleavage of the β-lactam ring in cephalosporins, which results in the expulsion of the leaving group at the 3′ position of the dihydrothiazine ring in the cephalosporin, has inspired the synthesis of various molecules for β-lactamase detection (*27*–*29*) and prodrug design (*30*, *31*). We have previously integrated a cephalosporin-based moiety into a hydrogel material that releases polystyrene beads upon β-lactamase–induced degradation (*32*, *33*). However, a hydrogel capable of β-lactamase–triggered antibacterial activity has not been previously demonstrated. Here, we introduce a system that employs a β-lactamase–cleavable cephalosporin crosslinker to directly couple bacterial enzymatic activity with antibiotic release, enabling infection-specific activation while minimizing drug exposure to healthy tissue.

In this study, we developed a β-lactamase–responsive antibacterial hydrogel system to target β-lactamase–producing bacteria. A key feature of this hydrogel is its ability to leverage β-lactamases as a selective trigger for the release of encapsulated ciprofloxacin, a broad-spectrum fluoroquinolone antibiotic that inhibits bacterial growth by interfering with DNA synthesis (*34*). To eliminate potential passive release of the antibiotic from the hydrogels, we loaded ciprofloxacin into liposomes before encapsulating the drug-loaded liposomes into the hydrogels. We conducted in vitro studies to assess the bacteria-triggered degradation of the hydrogels and release of ciprofloxacin and in vivo studies to validate the efficacy of the hydrogel in the treatment of a murine wound infection while comparing to a clinically used gold standard treatment. We also conducted ex vivo studies to examine antibacterial efficacy in a porcine burn infection model along with potential for antibiotic resistance development. Our findings suggest that these bacterial enzyme-responsive hydrogels have the potential to provide targeted, on-demand infection eradication while minimizing unnecessary exposure to antibiotics. By releasing the antibiotic only in the presence of β-lactamase–producing bacteria, our hydrogel system provides effective treatment while minimizing susceptibility to antibiotic resistance.

## RESULTS

### Β-Lactamase–responsive antibacterial hydrogel fabrication

**Fig. 1.**
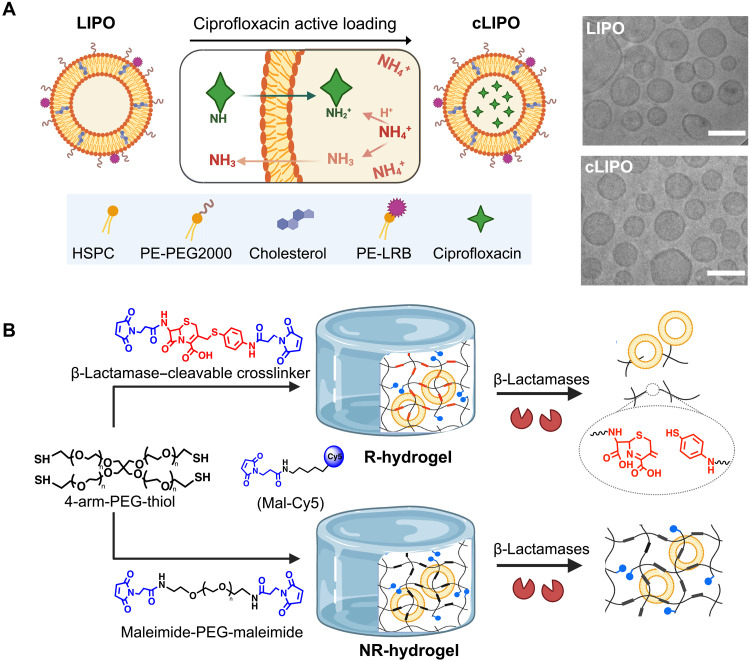
Antibacterial β-lactamase–responsive hydrogel formulation. (**A**) Schematic of liposomal cargo encapsulated in β-lactamase–responsive hydrogels. Liposomes (LIPOs) were formulated with a phospholipid-cholesterol bilayer, decorated with PEG to enhance stability, and labeled with LRB for fluorescence tracking. Ciprofloxacin-loaded liposomes (cLIPOs) were prepared using a transmembrane ammonium gradient active loading approach. Created in BioRender. A. Abbasi (2025); https://BioRender.com/yvmx6si. Representative cryo-TEM images of LIPOs and cLIPOs. Scale bars, 100 nm. (**B**) β-Lactamase–responsive (R) and nonresponsive (NR) hydrogels were prepared via a thiol-maleimide crosslinking reaction. R-hydrogels degrade in the presence of β-lactamases to enable triggered cargo release. Bismaleimide-terminated cephalosporin and PEG derivatives were used as crosslinkers for R- and NR-hydrogels, respectively. Both hydrogel formulations were labeled with Cy5 for fluorescent monitoring of hydrogel degradation. Created in BioRender. A. Abbasi (2025); https://BioRender.com/btvyxxf.

To develop bacteria-responsive hydrogels that release antimicrobial agents on-demand, we first prepared ciprofloxacin-loaded liposomes (cLIPOs), which were subsequently encapsulated into β-lactamase–responsive hydrogels. Nondrug-loaded liposomes (LIPOs) were formulated using thin-film hydration and extrusion (*35*) with 69.9% (w/w) hydrogenated soy phosphatidylcholine (HSPC), 20% (w/w) cholesterol, 10% (w/w) 1,2-distearoyl-*sn*-glycero-3-phosphoethanolamine-*N*-(methoxy(polyethylene glycol)-2000) (PE-PEG2000), and 0.1% (w/w) PE-(lissamine rhodamine B) (LRB); LRB was included to enable in situ monitoring. Ciprofloxacin, an amphipathic weak base, was actively loaded into the aqueous core of LIPOs using a transmembrane ammonium gradient (Fig. 1A) (*36*). The successful fabrication of LIPOs and cLIPOs was confirmed using cryo–transmission electron microscopy (cryo-TEM), which revealed spherical vesicles with distinct lipid bilayers surrounding aqueous cores, characteristic of unilamellar liposomes (Fig. 1A). Dynamic light scattering indicated a hydrodynamic diameter of ~105 nm for both drug- and nondrug-loaded liposomes (fig. S1, A and B), while zeta (ζ)-potential measurements showed that both formulations exhibited a net neutral charge (fig. S1C). We found that cLIPOs exhibited antibacterial activity against both Gram-negative and Gram-positive bacteria, namely, *P. aeruginosa* Xen41 and *S. aureus* Xen21, with minimum inhibitory concentrations (MICs) comparable to or slightly below the free ciprofloxacin MIC of ~0.3 μg/ml (fig. S1D). This observation is consistent with previous reports of liposomal ciprofloxacin formulations, where encapsulation improved drug stability and enhanced interaction with bacterial membranes, thereby facilitating more efficient local delivery at the cell surface (*37*). The encapsulation efficiency (EE) of ciprofloxacin was ~88%, with a drug loading of ~8% (w/w) (fig. S1E).

Following the successful fabrication and characterization of the antibacterial cargo, we proceeded to develop the hydrogels. We first synthesized a β-lactamase–cleavable crosslinker by functionalizing a cephalosporin derivative [7-amino-3-chloromethyl-3-cephem-4-carboxylic acid *p*-methoxybenzyl ester hydrochloride (ACLE)] with terminal maleimide groups (fig. S2A) (*32*, *33*). Successful synthesis was confirmed via proton nuclear magnetic resonance (^1^H NMR) and mass spectrometry (fig. S2, B and C). We then prepared the β-lactamase–responsive (R-) hydrogels by reacting 4-arm PEG-thiol macromers with this cleavable crosslinker through a Michael-type thiol-maleimide addition reaction (Fig. 1B). Nonresponsive (NR-) hydrogels were also prepared using a maleimide-functionalized PEG crosslinker in place of the β-lactamase–cleavable crosslinker. To enable in situ monitoring of the hydrogel, we labeled the PEG backbone of both hydrogel formulations with a Cy5 fluorophore by incorporating a maleimide-functionalized Cy5 during hydrogel crosslinking (Fig. 1B). Both R- and NR-hydrogels exhibited less than 6% unreacted thiols upon hydrogel fabrication, indicating efficient crosslinking and fluorophore labeling (fig. S3A). In addition, both hydrogels exhibited comparable equilibrium swelling ratios and elastic moduli (fig. S3, B and C). Scanning electron microscopy (SEM) revealed a similar porous structure for both formulations (fig. S3, D and E).

Liposomes were incorporated into the hydrogels by suspending them in the 4-arm PEG-thiol macromer solution during hydrogel fabrication. After crosslinking, fluorescence imaging confirmed homogeneous distribution of cLIPOs within the hydrogel matrix (fig. S4). No significant difference was observed in the elastic modulus between liposome-loaded (both cLIPOs and LIPOs) and nonliposome-loaded R-hydrogels, indicating that the incorporation of liposomes did not affect responsive hydrogel gelation (fig. S5). The stability of cLIPOs within the R-hydrogels was evaluated by incubating the hydrogels in 1× phosphate-buffered saline (PBS) at pH 7 and pH 4 at 25°C, with the acidic pH aimed at solubilizing any potentially aggregated ciprofloxacin within the hydrogel. Absorbance measurements after 7 days indicated no detectable ciprofloxacin release into the incubation buffer suggesting that cLIPOs remained stable within the hydrogels over this period (fig. S6).

### Triggered degradation, drug release, and cytocompatibility of β-lactamase–responsive hydrogels

**Fig. 2.**
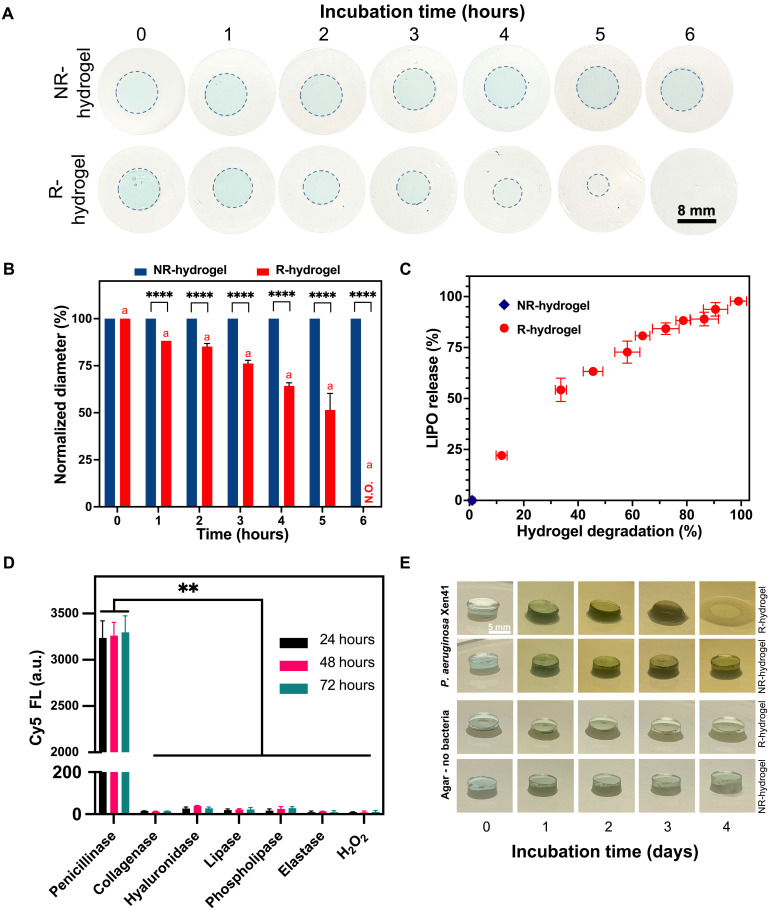
β-lactamase-triggered degradation of R-hydrogels in vitro. (**A**) Representative images of R- and NR-hydrogels incubated in 1× PBS containing β-lactamases from *P. aeruginosa* (50 U/ml) over time. Dashed outlines mark the hydrogel circumference at each time point. Hydrogels appear blue due to Cy5 labeling incorporated into the polymer network, which was used for fluorescence tracking. (**B**) Diameter of R-hydrogels decreased over time when incubated in the β-lactamase solution, while the diameter of NR-hydrogels remained unchanged (N.O., not observed). (**C**) β-Lactamase–triggered liposome release and hydrogel degradation kinetics. Release of LIPOs was measured by examining LRB fluorescence, while hydrogel degradation was measured by examining Cy5 fluorescence in the incubation media of R-hydrogels and NR-hydrogels incubated with β-lactamases. Each data point corresponds to a time point at 30-min intervals. R-hydrogel incubation media showed time-dependent increases in both LRB and Cy5 fluorescence (FL) signals, while NR-hydrogel incubation media showed no detectable FL, indicating hydrogel stability. (**D**) Cy5 FL of incubation media following R-hydrogel incubation with wound-associated enzymes, hydrogen peroxide (as a common ROS present in wounds), and penicillinases (i.e., β-lactamases) from *B. cereus.* Only penicillinases induced significant Cy5 FL, confirming the specificity of R-hydrogel degradation. (**E**) R-hydrogels degraded when incubated on agar inoculated with *P. aeruginosa* Xen41 (β-lactamase producing) but not on sterile agar (no bacteria). NR-hydrogels remained unchanged in both conditions. All digital camera images are representative of four independent experiments. Data are presented as average ± SD (*n* > 3). Statistical analysis was performed using two-way analysis of variance (ANOVA) with Tukey’s post hoc analysis. Matching letters indicate a significant difference in normalized diameter of each hydrogel at all time points (*P* < 0.05), and ****P < 0.0001 indicates a significant difference between hydrogel formulations at a given time point. **P < 0.01 indicates significant differences in Cy5 FL between enzyme/ROS groups.

The β-lactamase responsiveness of R-hydrogels was examined in vitro by incubating R- and NR-hydrogels in a solution of β-lactamases, specifically cephalosporinases from *P. aeruginosa*. Visual observations revealed a substantial reduction in R-hydrogel size over time, with no discernable hydrogel remaining by 6 hours, indicating β-lactamase–triggered hydrogel degradation (Fig. 2A). In contrast, NR-hydrogels showed no noticeable change in size over the same time, confirming their lack of responsiveness to β-lactamases (Fig. 2A). These observations were supported by quantification of hydrogel diameter in these conditions (Fig. 2B).

We further examined hydrogel degradation and the release of encapsulated LIPOs by monitoring the fluorescence intensities of the hydrogel backbone tag, Cy5, and the liposome tag, LRB, in the β-lactamase incubation media (Fig. 2C). The increasing fluorescence of both Cy5 and LRB over time confirmed R-hydrogel degradation and the consequent release of the encapsulated LIPOs. As hypothesized, no fluorescence signal for Cy5 or LRB was detected for NR-hydrogels, indicating their stability at these conditions with no release of hydrogel backbone components or liposomes. We estimated the ciprofloxacin release profile from R-hydrogels by correlating the release kinetics of the LIPOs with the known ciprofloxacin loading of cLIPOs within cLIPO-loaded R-hydrogels (i.e., 7 μg per hydrogel). As shown in fig. S7, the encapsulated ciprofloxacin (~1.5 μg/ml) was estimated to release within the first 30 min of β-lactamase exposure, which exceeds the MIC of ciprofloxacin against *P. aeruginosa* Xen41 fivefold.

To investigate the β-lactamase specificity of R-hydrogels, we exposed R-hydrogels to various enzymes commonly found in wounds, including collagenases, hyaluronidases, lipases, elastases, and phospholipases (*38*). Furthermore, to assess the impact of ROS, which are elevated during wound healing (*39*), R-hydrogels were incubated in a hydrogen peroxide (H_2_O_2_) solution. Having already confirmed R-hydrogel degradation in response to cephalosporinases from *P. aeruginosa*, we also evaluated their response to penicillinases from *Bacillus cereus*. Hydrogels were incubated in 1× PBS containing the enzymes of interest, H_2_O_2_, or penicillinases, and the fluorescence intensity of Cy5 in the incubation media was measured as an indicator of hydrogel degradation. After 24 hours, the media containing penicillinases exhibited a marked increase in Cy5 fluorescence, correlating with the complete degradation of the R-hydrogels, again confirming β-lactamase responsiveness (Fig. 2D). In contrast, the Cy5 fluorescence intensity remained consistently low (near autofluorescence levels) in the PBS containing the other enzymes and H_2_O_2_, even after 72 hours of incubation (Fig. 2D), with no visible change in hydrogel size. These results highlight the specificity of R-hydrogel degradation by β-lactamases.

Having established the β-lactamase triggered degradation and specificity of R-hydrogels, we next examined their behavior in the presence of β-lactamase and non–β-lactamase–producing bacteria. Specifically, we used *P. aeruginosa* Xen41 and *S. aureus* Xen29, which were confirmed as β-lactamase–producing and non–β-lactamase–producing strains, respectively, via a nitrocefin hydrolysis assay (fig. S8). R- and NR-hydrogels were incubated on *P. aeruginosa* Xen41 and *S. aureus* Xen29 agar cultures. As shown in Fig. 2E, R-hydrogels exhibited clear degradation over time in the presence of *P. aeruginosa* Xen41, while NR-hydrogels remained stable. In contrast, both R- and NR-hydrogels remained unchanged when exposed to *S. aureus* Xen29, over the 4 days examined as shown in fig. S9, comparable to hydrogels incubated on sterile, nonbacteria-inoculated agar for the same duration (Fig. 2E). These results confirm that R-hydrogels undergo selective degradation in the presence of β-lactamase–producing bacteria, while NR-hydrogels remain unchanged under these conditions.

Before investigating R-hydrogel behavior in vivo, we assessed their cytocompatibility by examining the effects of the hydrogel degradation products on murine fibroblast metabolic activity and human red blood cell (RBC) lysis. R-hydrogels loaded with cLIPOs and LIPOs were first completely degraded using cephalosporinases from *P. aeruginosa*. Fibroblasts and RBCs were then exposed to these degradation products. Controls of β-lactamases, LIPOs, and cLIPOs in PBS at concentrations equivalent to those expected from fully degraded hydrogels were also tested. No reduction in cell viability was observed in any of the treatments compared to untreated controls (fig. S10A). In addition, no hemolytic activity was observed compared to positive controls treated with Triton X-100 (fig. S10B). These findings indicate that both the hydrogel degradation products and their individual components exhibit excellent cytocompatibility.

### β-Lactamase–responsive hydrogel performance in a murine skin abrasion wound infection

**Fig. 3.**
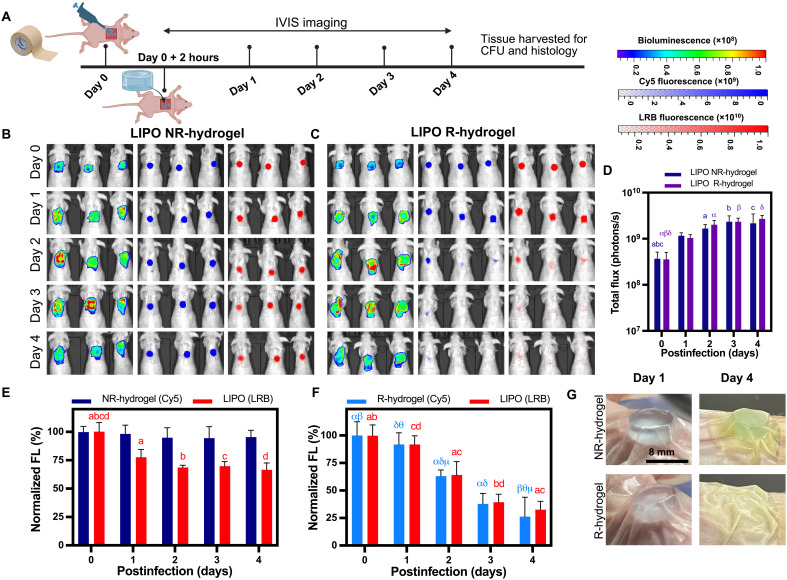
In vivo degradation of fluorescently labeled LIPO-loaded R-hydrogels (nondrug loaded) in a murine skin abrasion infection model. (**A**) Schematic overview of the in vivo study timeline. Created in BioRender. A. Abbasi (2025); https://BioRender.com/px31h52. Superficial skin wounds were created via tape-stripping, leading to the removal of a large portion of the epidermal layer, followed by inoculation with *P. aeruginosa* Xen41 and subsequent application of hydrogels. Bacterial bioluminescence as well as Cy5 (representing hydrogel degradation products) and LRB (representing fluorescently labeled, nondrug-loaded liposomes used to monitor the cargo release) FL were monitored noninvasively using IVIS imaging daily for 4 days postinfection. Representative IVIS images over time monitoring *P. aeruginosa* Xen41 bioluminescence (left), Cy5 FL (blue, middle), and LRB FL (red, right) for mice receiving (**B**) nondrug-loaded LIPO-loaded NR-hydrogels and (**C**) nondrug-loaded LIPO-loaded R-hydrogels (*n* = 5 mice per group). (**D**) Quantification of bacterial bioluminescence over time following application of LIPO-loaded R- and NR-hydrogels at the infection site. Cy5 FL and LRB FL over time following application of (**E**) LIPO-loaded NR-hydrogels and (**F**) LIPO-loaded R-hydrogels at the infection site. (**G**) Representative digital camera images of wound sites posttreatment showing the degradation of the LIPO-loaded R-hydrogels on an infected wound compared to a stable LIPO-loaded NR-hydrogel on an infected wound. Data are presented as average ± SD (*n* = 5). Statistical analysis was performed using two-way ANOVA with Tukey’s post hoc analysis. Matching letters indicate a significant difference in bioluminescence flux or FL signal per hydrogel formulation at across time points (*P* < 0.05).

Next, we examined the efficacy of R-hydrogels in a murine skin abrasion wound infection model (Fig. 3A). A dorsal wound (1 cm by 1 cm) was created by repeated tape stripping in anesthetized immunocompetent mice, resulting in visible skin reddening and glistening with no observed bleeding (fig. S11A). The effectiveness of the tape stripping method was confirmed via histological examination (fig. S11B), which revealed an 85% decrease in epidermal thickness following nine tape applications (fig. S11C), indicating successful creation of a superficial wound. Once the wounds were successfully established, the β-lactamase–producing bacteria, *P. aeruginosa* Xen41 [10^7^ colony forming units (CFU)] was applied to the wound site. After 2 hours, treatments were applied to the wound site and infection progression was monitored noninvasively via in vivo imaging system (IVIS) bioluminescence imaging over 4 days postinfection (Fig. 3A). Wound tissue was harvested for CFU enumeration and histology on the fourth day.

Before assessing the antibacterial efficacy of cLIPO-loaded R-hydrogels in this wound infection model, we examined infection progression along with the degradation behavior of fluorescently labeled, nondrug-loaded, LIPO-loaded R- and NR-hydrogels. These LIPOs labeled with LRB were not loaded with ciprofloxacin, serving solely to monitor cargo release. In addition to daily monitoring of *P. aeruginosa* Xen41 bioluminescence, we measured Cy5 fluorescence (representing the hydrogel matrix) and LRB fluorescence (representing fluorescently labeled liposomes to monitor cargo release) (Fig. 3B). The bioluminescence signal from the infected wounds in both the R- and NR-hydrogel group increased steadily and remained localized to the wound site, confirming the successful establishment and persistence of the bacterial infection over the 4-day study (Fig. 3, B and C). No significant differences in bacterial bioluminescence flux were observed between the groups at any time point (Fig. 3D), as expected since no antibiotic cargo was present in these formulations. NR-hydrogels applied to infected wounds showed no significant change in Cy5 signal over time, indicating that these hydrogels remained stable as expected for hydrogels formed without the β-lactamase–cleavable crosslinker (Fig. 3, B and E). The LRB signal in these NR-hydrogels decreased slightly initially, followed by no change after the first day, indicating potential release of surface associated liposomes upon hydrogel application (Fig. 3, B and E). In contrast, R-hydrogels encapsulating fluorescently labeled LIPOs applied to infected wounds exhibited degradation, facilitating the release of LIPOs into the wound environment. This triggered release was confirmed by a decrease in Cy5 and LRB fluorescence intensities at the infection site over time (Fig. 3, C and F). On day 4 postapplication, distinct differences in hydrogel appearance and integrity were observed for R-hydrogels versus NR-hydrogels (Fig. 3G). NR-hydrogels remained largely intact on the infected wounds, maintaining their original size and shape. In contrast, R-hydrogels were difficult to visualize due to a large decrease in size and change in shape, suggesting degradation in response to the wound infection.

**Fig. 4.**
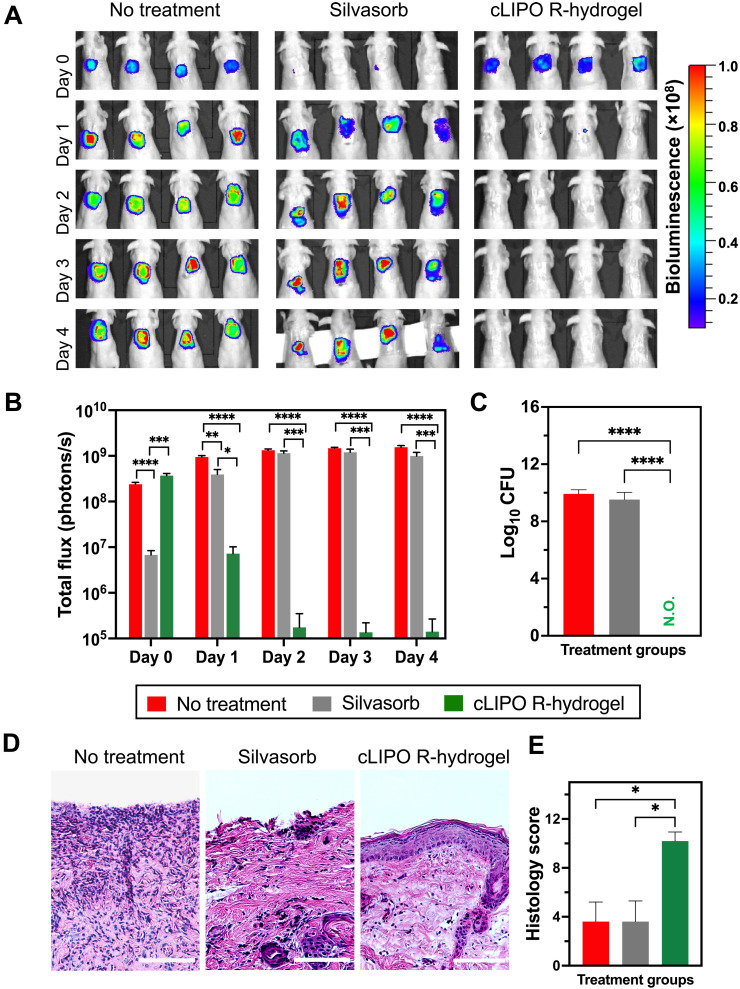
In vivo degradation and drug release from cLIPO-loaded R-hydrogels in a murine skin infection model. (**A**) Representative IVIS images showing bacterial bioluminescence at different time points following treatment with cLIPO-loaded R-hydrogel or Silvasorb hydrogel. (**B**) Quantification of bacterial bioluminescence over four days post-treatment. By day 2, the bacterial bioluminescence signal in the cLIPO-loaded R-hydrogel group decreased to near-background levels. Data are presented as average ± SD (*n* = 8). Statistical analysis was performed using two-way ANOVA with Tukey’s post hoc analysis (*P < 0.05, **P < 0.01, ***P < 0.001, and ****P < 0.0001). (**C**) Enumeration of CFUs from total wounded skin tissue on day 4 post-treatment (N.O., not observed). Data are presented as average ± SD (*n* = 4). Statistical analysis was performed using one-way ANOVA with Tukey’s post hoc analysis (****P < 0.0001). (**D**) Representative histological sections of wound biopsies stained with H&E. Scale bars, 100 μm. (**E**) Blinded histology scoring presented as average ± SD (*n* = 5). Statistical analysis was performed using one-way ANOVA with Tukey’s post hoc analysis (*P < 0.05).

Once we confirmed that the bacteria-responsive properties of the R-hydrogels were maintained in vivo, we proceeded to examine the antibacterial efficacy of cLIPO-loaded R-hydrogels on infected wounds. The activity of these hydrogels was compared to Silvasorb, a clinically used antibacterial hydrogel containing ionic silver as the encapsulated antimicrobial agent. IVIS imaging conducted 2 hours posttreatment (i.e., day 0) revealed a significantly lower bacterial bioluminescence for wounds treated with Silvasorb compared to untreated and cLIPO-loaded R-hydrogel treated wounds, which exhibited comparable levels of *P. aeruginosa* bioluminescence at this time (Fig. 4, A and B). However, at day 1 posttreatment, the cLIPO-loaded R-hydrogels demonstrated a large reduction in bacterial bioluminescence, reaching a level comparable to that which was observed for the Silvasorb-treated group on day 0 (Fig. 4, A and B). Conversely, the Silvasorb-treated group experienced a resurgence in bacterial bioluminescence by day 1 posttreatment, which remained elevated over the following 3 days, mirroring the untreated control infection group. By day 2 posttreatment, the bacterial bioluminescence signal in the cLIPO-loaded R-hydrogel treated group decreased to near background levels (Fig. 4B).

The cLIPO-loaded R-hydrogel group showed no signs of infection recurrence throughout the four-day study, indicating prolonged and robust antibacterial activity. This finding was further validated by the CFU counts from tissue harvested on day 4 posttreatment (Fig. 4C). Colony enumeration revealed that the bacterial burden in the Silvasorb-treated group was comparable to mice in the untreated control group, highlighting the inability of Silvasorb to prevent infection recurrence following an initial rapid reduction in bacteria upon application. In contrast, the R-hydrogel group achieved complete clearance of the infection, with no detectable CFUs in the tissue harvested at day 4.

Hematoxylin and eosin (H&E) staining of wound biopsies revealed differences in tissue integrity and healing among the treatment groups 4 days postinfection (Fig. 4D). Both the no treatment infected control group and the Silvasorb-treated group exhibited tissue damage. In contrast, the cLIPO-loaded R-hydrogel treatment group showed clear signs of infection clearance and reepithelization at the wound site, confirming that effective eradication of the infection allowed for effective wound repair. Immunofluorescence imaging of macrophages at the wound site further supported these findings by demonstrating reduced macrophage infiltration at the wound site in the cLIPO-loaded R-hydrogel–treated group at day 4 compared to the other treatment groups (fig. S12). To quantitatively assess the overall healing progress of infected wounds, a blinded scoring system was used. The scoring system, detailed in table S1, ranged from 0 (open and unhealed wound) to 12 (completely healed wound), with higher scores indicating more advanced stages of healing (*40*). As shown in Fig. 4E, the cLIPO-loaded R-hydrogel–treated group achieved significantly higher histology scores compared to the untreated controls and Silvasorb-treated groups that exhibited comparable histology scores.

### Antibacterial activity of β-lactamase–responsive antibacterial hydrogels in an ex vivo burn infection model

**Fig. 5.**
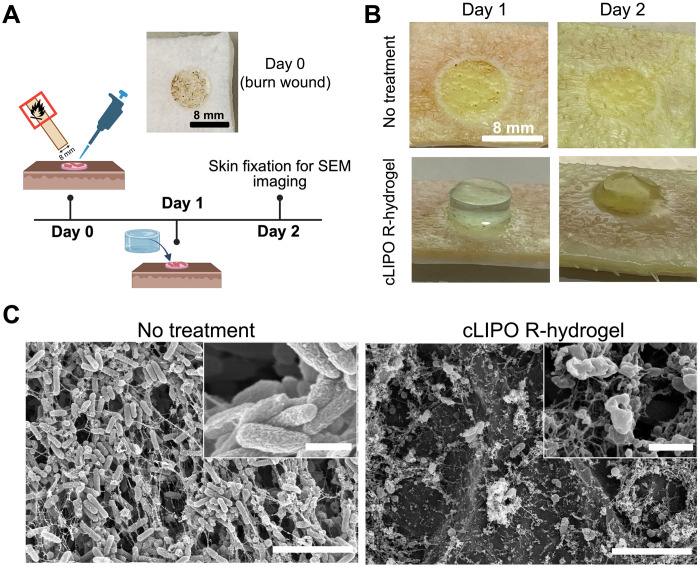
β-lactamase–triggered hydrogel degradation and drug release in an ex vivo burn infection model. (**A**) Schematic overview of the ex vivo study timeline forming an 8-mm-diameter burn wound on porcine skin. Created in BioRender. A. Abbasi (2025); https://BioRender.com/gvjqyrn. (**B**) Digital photographs of cLIPO-loaded R-hydrogels applied to porcine skin inoculated with *P. aeruginosa* Xen41. Untreated wounds served as a control. All digital camera images are representative of four independent experiments. (**C**) SEM images of *P. aeruginosa* Xen41–infected porcine skin burn wounds with and without cLIPO-loaded R-hydrogel treatment. Scale bars, 5 μm. Insets show the bacterial morphologies with scale bars = 1 μm.

After demonstrating the complete eradication of *P. aeruginosa* through the application of cLIPO-loaded R-hydrogels in a murine skin abrasion wound infection model, we further explored hydrogel application in an ex vivo porcine skin burn wound infection model. Ex vivo infected burn wounds were created by briefly placing a heated 8-mm-diameter copper rod on porcine skin tissue, followed by inoculation with 10^7^ CFU of either the β-lactamase–producing *P. aeruginosa* Xen41 or the non–β-lactamase–producing control, *S. aureus* Xen29 (Fig. 5A). One day postwounding and inoculation, cLIPO-loaded R-hydrogels were applied to the infected burn wounds. After a 24-hour incubation at 37°C, R-hydrogels degraded on wounds inoculated with *P. aeruginosa* (Fig. 5B) while remaining unchanged on wounds inoculated with *S. aureus* Xen29 (fig. S13A), consistent with our agar studies (Fig. 2E).

The subsequent SEM analysis of the ex vivo burn wounds revealed robust eradication of *P. aeruginosa* at 1 day posttreatment with cLIPO-loaded R-hydrogels compared to untreated wounds (Fig. 5C). This substantial antimicrobial activity can be attributed to the triggered degradation of R-hydrogels and release of the encapsulated antibacterial liposomes, driven by β-lactamases production by *P. aeruginosa* Xen41. SEM images showed that any *P. aeruginosa* bacteria remaining after hydrogel treatment exhibited significant morphological damage. In contrast, for *S. aureus* Xen29–inoculated burn wounds that were treated with cLIPO-loaded R-hydrogels, a robust *S. aureus* biofilm was observed at day 1. *S. aureus* cells exhibiting smooth cell walls and visible extracellular polymeric substances, forming the biofilm matrix, were observed similar to what was seen for untreated controls (fig. S13B). These results are attributed to the selective β-lactamase–triggered antimicrobial activity of the hydrogels, which was not activated by the non–β-lactamase–producing *S. aureus* Xen29.

### Assessing ciprofloxacin resistance development in the presence of β-lactamase–responsive antibacterial hydrogels

**Fig. 6.**
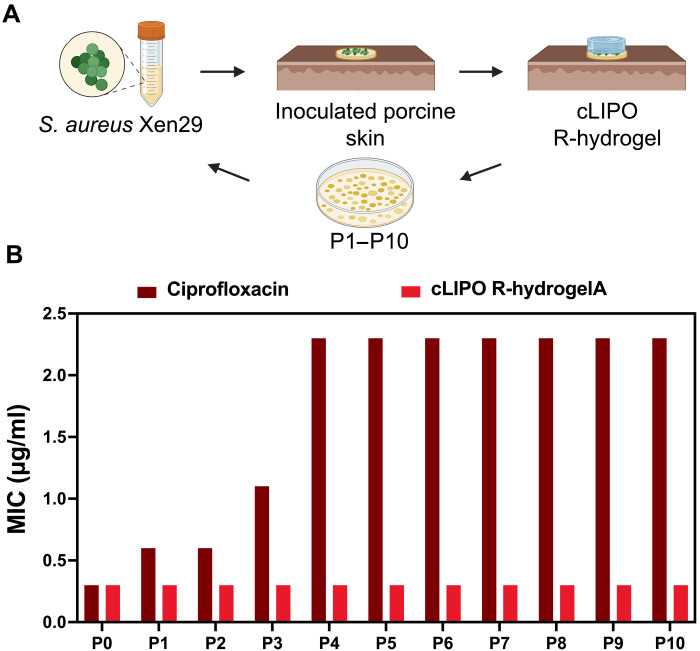
Evaluating resistance development in *S. aureus* Xen29 after serial exposure to cLIPO-loaded R-hydrogels. (**A**) Schematic illustrating the procedure of exposing *S. aureus* Xen29 bacteria (non–β-lactamase producing) to R-hydrogels loaded with cLIPOs on ex vivo burn wound infections. Created in BioRender. A. Abbasi (2025); https://BioRender.com/x1s4ryh. Following daily application of the hydrogel, bacteria was collected and cultured. The ciprofloxacin MIC against *S. aureus* Xen29 was evaluated, and this cultured bacteria was used to inoculate porcine burn wounds, which were again treated with cLIPO-loaded R-hydrogels. This process was repeated for ten cycles. (**B**) Graph compares the MIC for ciprofloxacin against *S. aureus* Xen29 at each cycle of exposure to the cLIPO-loaded R-hydrogels versus the MIC of *S. aureus* Xen29 cultured in the presence of subtherapeutic doses of free ciprofloxacin over 10 cycles (0.1 μg/ml; approximately one-third of the MIC of ciprofloxacin susceptible *S. aureus* Xen29).

Last, we investigated whether ciprofloxacin-susceptible, non–β-lactamase–producing bacteria would develop resistance to ciprofloxacin over a prolonged exposure to cLIPO-loaded R-hydrogels. Ex vivo porcine burn wounds inoculated with *S. aureus* Xen29 were treated with these hydrogels over a period of 10 days, simulating a scenario where hydrogels are applied as a prophylactic measure and remain intact on the skin (Fig. 6A). Each day, *S. aureus* from the skin was cultured and the ciprofloxacin MIC was determined to investigate any potential resistance development due to subtherapeutic drug exposure, possibly resulting from surface-exposed liposomes or passive diffusion. The cultured *S. aureus* was used to inoculate fresh burn wounds. Despite 10 consecutive 24-hour hydrogel exposures, no increase in ciprofloxacin MIC against *S. aureus* Xen29 was observed (Fig. 6B). As observed in the in vitro stability test of cLIPO-loaded R-hydrogels in PBS (fig. S6), this result further confirms that intact R-hydrogels applied to the *S. aureus*–inoculated ex vivo burn wounds do not passively release ciprofloxacin or do so at a negligible concentration insufficient to drive resistance. In sharp contrast, *S. aureus* Xen29 suspension cultures exposed to sub-MIC levels of free ciprofloxacin (i.e., 0.1 μg/ml, which is approximately one-third of the ciprofloxacin MIC against *S. aureus* Xen29) over the same duration as the hydrogel studies, rapidly developed resistance. The MIC increased gradually over the first three passages, and by the fourth passage, the MIC had increased eightfold. Thus, while sub-MIC ciprofloxacin readily selected for resistant mutants, the enzyme-responsive hydrogels did not create conditions that promote resistance development.

## DISCUSSION

While antibiotics remain the primary treatment for wound infections, their efficacy is highly dependent on maintaining effective drug concentrations at the infection site (*41*). It is widely recognized that the bactericidal activity of antibiotics is concentration dependent, with higher concentrations achieving optimal therapeutic outcomes, while subinhibitory levels can promote the development of drug resistance and recurrence of infections (*41*). Now, health care systems worldwide are struggling with the uncontrolled spread of antibiotic-resistant pathogens, exacerbated by the misuse and overuse of antibiotics. This misuse creates selective pressure that favors the survival and propagation of resistant bacterial strains (*42*, *43*). In this challenging context, biomaterial-based antibacterial therapies have emerged as a promising alternative to traditional antibiotic treatments (*44*). Among these biomaterials, hydrogels show great potential for localized and controlled drug delivery in infection and wound management (*32*, *45*, *46*). In addition to the advantages of the water-swollen hydrogel network for wound healing, they provide a versatile platform for encapsulating a wide range of therapeutics—including small-molecule drugs, nanoparticles, and even mammalian cells—while offering customizable release profiles, sustained or triggered and tailored to specific therapeutic needs (*46*, *47*). This adaptability makes hydrogels a potentially powerful tool to address the complex challenges posed by antibiotic resistance and wound infections.

To meet the demand for advanced drug delivery systems that provide precise, controlled antibiotic release at the site of infection, here, we developed bacteria-responsive hydrogels capable of delivering effective drug concentrations on-demand for the treatment of wound infections. This approach helps to minimize systemic drug exposure and reduces the potential for resistance development. We leveraged the β-lactam hydrolysis activity of bacterial β-lactamases to trigger liposomal antibiotic release from these responsive hydrogels. We integrated a modified cephalosporin derivative into a PEG hydrogel matrix, creating the β-lactamase–responsive hydrogels. We also produced a nonresponsive PEG hydrogel control formulation that was used to compare with our R-hydrogel formulations across all studies. Upon hydrogel formation, R- and NR- hydrogels exhibited similar physical properties, including elastic moduli, crosslinking efficiency, and swelling ratio, suggesting that the β-lactam ring did not interfere with the crosslinking process of the R-hydrogels.

In this study, we selected ciprofloxacin as the antibiotic of interest. This fluoroquinolone antibiotic is used in the treatment of a wide range of infections, including skin and soft tissue infections caused by *P. aeruginosa* and *S. aureus* (*48*). To ensure a truly enzyme-responsive release, we encapsulated the antibiotic within liposome nanocarriers that were subsequently entrapped within the hydrogel matrix. The transmembrane ammonium gradient method was used to actively incorporate the hydrophilic ciprofloxacin molecules into the liposomes, enhancing the loading capacity compared with passive encapsulation methods (*36*, *49*–*51*). The active loading approach has also been observed to lead to excellent drug entrapment and liposome stability (*52*), which was also observed in our study, where negligible passive diffusion of ciprofloxacin was observed from the R-hydrogel matrix in PBS lacking β-lactamases. Our in vitro studies demonstrated effective degradation of R-hydrogels and release of encapsulated cargo from R-hydrogels in the presence of β-lactamases and β-lactamase–producing bacteria. Based on the LIPOs release studies and the EE of ciprofloxacin in cLIPOs, we estimated that within 30 min of exposure to β-lactamases, ciprofloxacin concentrations far exceeding the MIC for *P. aeruginosa* Xen41 would be released. Moreover, the total antibiotic dose released can be further tuned by adjusting liposome loading based on different therapeutic needs. The stability of these hydrogels in the presence of non–β-lactamase enzymes and ROS, combined with their noncytotoxic and nonhemolytic activity, along with the on-demand ciprofloxacin delivery underscores the potential of this intelligent, responsive antibiotic delivery system.

In vivo studies using a murine skin abrasion wound infection model corroborated our in vitro results, demonstrating that R-hydrogels undergo degradation and release of the antibiotic payload in response to *P. aeruginosa* Xen41 infection. A single application of cLIPO-loaded R-hydrogels completely eradicated the infection. In contrast, treatment with a clinically used hydrogel dressing containing ionic silver showed only a transient bacterial reduction, followed by rapid infection resurgence. While silver has been shown to exhibit antibacterial activity (*53*), these results highlight the superior efficacy of R-hydrogels in managing wound infections. Notably, in mice treated with cLIPO-loaded R-hydrogels, the epidermis layer was fully regenerated, demonstrating the hydrogel capacity for promoting wound healing. Histological analysis also revealed a reduced presence of macrophages in the wound area of R-hydrogel–treated wounds. Although macrophages are critical for early wound response, excessive accumulation can impair healing through increased proteolysis, matrix degradation, and inducing cellular senescence (*54*, *55*). Overall, histological evaluation confirmed that R-hydrogels not only suppress infection more effectively than silver-based dressings but also promoted superior wound healing and resolution of inflammation. We further demonstrated the versatility of R-hydrogels by examining their use in an ex vivo porcine burn wound infection. Electron microscopy showed that untreated *P. aeruginosa* infected burn wounds exhibited a robust biofilm formation, while a complete lack of biofilm and the presence of only damaged bacterial cells was observed for R-hydrogel–treated wounds. These results are especially promising, given the prevalence of biofilms in wound infections and the notable recalcitrance of these microbial communities (*56*).

Antimicrobial biomaterials under development must also be evaluated for their potential to mitigate or promote antimicrobial resistance in the host microbiota. In this study, we evaluated whether ciprofloxacin-loaded R-hydrogels could induce resistance when applied to wounds without undergoing degradation-triggered release of the encapsulated antimicrobial cargo. This scenario represents a prophylactic application of R-hydrogels to prevent the development of severe infection caused by β-lactamase producing bacteria. A key concern in this situation is whether exposure to intact R-hydrogels could induce ciprofloxacin resistance in commensal wound bacteria. To investigate this possibility, we applied R-hydrogels on ex vivo porcine burn wounds inoculated with *S. aureus* Xen29, a non–β-lactamase–producing strain used as a model for commensal staphylococci (*57*). This strain is known to rapidly develop ciprofloxacin resistance under subinhibitory exposure. After 10 days of exposure to the cLIPO-loaded R-hydrogels, no resistance development was observed, in contrast to the rapid resistance development we observed for *S. aureus* Xen29 exposed to subinhibitory concentrations of free ciprofloxacin. These findings suggest that the inherent stability of R-hydrogels and their liposomal payload minimizes the risk of driving antibiotic resistance. By avoiding the selective pressures typically associated with passive antibiotic release, this strategy that ensures triggered bacteria-specific release, offers a potential advantage for long-term wound management where lengthy or repeated applications of hydrogel dressings are often required.

Responsiveness to β-lactamase activity offers a distinct advantage in specificity for our hydrogel design, as these enzymes are produced by bacterial pathogens but not by mammalian cells or healing tissue, thereby minimizing unintended drug release in uninfected environments. β-lactamase–producing bacteria such as *P. aeruginosa*, *K. pneumoniae*, and methicillin-resistant *S. aureus* are among the most clinically relevant wound pathogens, particularly in chronic and multidrug-resistant infections where conventional therapies often fail. While the prevalence of β-lactamase–producing isolates in wound infections is well documented, quantitative measurements of enzyme activity in wound exudates remain limited. In our in vitro studies, we used β-lactamases (i.e., cephalosporinases from *P. aeruginosa*) at a concentration of 50 U/ml, consistent with the β-lactamase activity levels reported in clinical isolates [e.g., total β-lactamase activity from *Acinetobacter baumannii* extracts has been reported to be ~31 U (*58*)]. The direct quantification of β-lactamase activity in wound fluids will be an important future direction to enable more precise calibration of infection-responsive hydrogel systems. The responsive hydrogels developed here could also potentially be adapted for real-time in situ detection of β-lactamase activity. While we have explored β-lactamase responsiveness due its relevance to a majority of wound infections, the design approach can be extended to other bacterial enzymes in future hydrogel systems to target other potential infections.

Polymicrobial wound infections are an important consideration in the clinical translation of emerging antimicrobial wound dressing technologies (*59*). Polymicrobial interactions are well documented in chronic wounds and can alter antibiotic efficacy and resistance dynamics (*60*–*62*). In a mixed infection where β-lactamase–producing species coexist with non–β-lactamase producers, the triggered release of antibiotics from R-hydrogels would expose both bacterial populations to ciprofloxacin. High local antibiotic concentrations may eradicate all susceptible species. While our studies with defined single-species infections enabled clear assessment of hydrogel responsiveness, future studies can use polymicrobial wound infection models to directly evaluate hydrogel performance in these more complex conditions. Additional strategies such as combinatorial antibiotic formulations (*63*, *64*) or dual enzyme–responsive hydrogel approaches (*65*) could be explored to further ensure broad-spectrum activity. Further species-level identification before and after treatment, using 16*S* ribosomal RNA sequencing and expanded culture-based methods, in these future studies will allow for a more complete understanding of pathogen and microbiome influences on the hydrogel efficacy.

The responsive hydrogels developed in this study have demonstrated several promising results, indicating strong potential for future use as an antibacterial hydrogel wound dressing. Translating our findings from laboratory models to clinical practice will require several additional steps. First, the studies conducted here were limited to the use of ciprofloxacin. We expect that the reported approach can be readily adapted to other antibiotics or combinations of antibiotics, which must be explored in future studies. Our current in vivo studies have focused on acute infection scenarios with immediate treatment application following bacterial inoculation. While this approach effectively assesses the treatment efficacy of our hydrogel, it does not fully address the challenges posed by chronic infections or the presence of bacterial biofilms (*66*). Developing chronic infection models, particularly in large animals that better mimic these clinical complexities, will be essential for a comprehensive evaluation of R-hydrogel efficacy. Further evaluation of the host immune response and hydrogel biocompatibility in these models will also be needed. In addition, the bacterial strains used in laboratory settings often differ from those involved in clinical infections, potentially having adapted to laboratory conditions and lost critical virulence factors necessary for survival in a host (*67*). Therefore, it is important to conduct studies with clinical isolates of relevant bacterial species, including *P. aeruginosa* and others, to ensure that the bacteria retain their clinical characteristics, allowing for a more realistic assessment of the hydrogel therapeutic potential. Last, the scalability of production, cost-effectiveness, and storage stability are pivotal factors that will determine the feasibility of translating these hydrogels into clinical practice.

In summary, we have developed a β-lactamase–responsive hydrogel drug delivery system with potent on-demand antibacterial activity, offering a promising alternative therapeutic strategy for treating wound infections. Looking ahead, the integration of multistimuli-responsive elements presents a compelling avenue for future hydrogel design, enabling systems that release not only antibiotics in response to specific microbial cues but also other therapeutic agents that promote wound healing and modulate immune responses (*12*). Continued advances in polymer chemistry, coupled with emerging 3D printing techniques, (*68*, *69*), offers opportunities to fabricate these multifunctional hydrogels with tailored architectures suited for the complex wound microenvironment. The hydrogel drug delivery system reported in this study is an important step toward the development of personalized, next-generation antimicrobial wound care solutions.

## MATERIALS AND METHODS

### Materials

Unless otherwise stated, all reagents were purchased from commercial sources. ACLE was purchased from AK Scientific (Union City, California). 3-maleimidopropionic acid was obtained from TCI Chemicals (Tokyo, Japan). 4-aminothiophenol (ATP), triethylamine (TEA), *N*-methylmorpholine (NMM), anhydrous dichloromethane (DCM), anhydrous dimethylformamide (DMF), ethyl acetate, dimethyl sulfoxide (DMSO), trifluoroacetic acid (TFA), anisole, diethyl ether, 1-[bis(dimethylamino)methylene]1H-1,2,3-triazolo[4,5-b]pyridinium 3-oxid hexafluorophosphate (HATU), *N*,*N*-diisopropylethylamine (DIPEA), thin-layer chromatography silica gel 60 on glass plates, Luria Bertani (Lennox) broth, β-lactamases from *B. cereus* and *P. aeruginosa*, collagenases from *Clostridium histolyticum*, and lipases from *Pseudomonas cepacia*, along with the fluorometric thiol quantitation kit, PBS (1×), Triton X-100, methanol, sodium chloride, ammonium sulfate, paraformaldehyde, ethanol, 4-arm-PEG-thiol (20 kDa), and LB were acquired from MilliporeSigma (St. Louis, Missouri). Phospholipase A2 was acquired from ProSpec (East Brunswick, New Jersey). Cy5-maleimide and 1,11-bismaleimido-triethylene glycol (mal-PEG-mal, 494.5 Da) were purchased from Broad Pharm (San Diego, CA). Deuterated solvents and acetone-d_6_ were obtained from Cambridge Isotope Laboratories (Andover, Massachusetts). Ciprofloxacin hydrochloride was purchased from Cayman (Ann Arbor, Michigan). Lipid components for nanoparticle synthesis—including HSPC, cholesterol, PE-PEG2000, and PE-LRB—were all purchased from Avanti Polar Lipids (Alabaster, Alabama). Dulbecco’s modified Eagle’s medium (DMEM) and fetal bovine serum (FBS), along with penicillin-streptomycin, were purchased from Gibco-BRL (Grand Island, NY), and Caisson Laboratories (Smithfield, UT), respectively. Cell Counting Kit-8 (CCK-8) and nitrocefin were obtained from Apex Bio (Houston, TX), and rabbit RBCs were obtained from Innovative Research (Novi, MI). NIH-3T3 fibroblast cells were procured from the American Type Culture Collection (ATCC; Manassas, VA). Bioluminescent bacteria, Xen41 (*P. aeruginosa* PA01) and Xen29 (*S. aureus* ATCC 12600), were sourced from PerkinElmer (Waltham, Massachusetts). Porcine skin tissue was obtained from the Brown University Center for Animal Resources and Education (Providence, RI). Anti-macrophage antibody RM0029-11H3 and Alexa 568 anti-rat immunoglobulin G were obtained from Abcam. Ultrapure deionized water (18.2 MΩ·cm, MilliporeSigma, Billerica, MA) was used in all experiments.

### General methods and instrumentation

^1^H-NMR spectra were recorded on a Bruker DRX Avance 400 MHz spectrometer with chemical shifts stated as δ in parts per million (ppm) using acetone-d_6_ (2.05 ppm) as the reference. High-resolution mass spectrometry using electrospray ionization (ESI) was performed on an Agilent 6530 liquid chromatograph tandem mass spectrometer (LC-MS). Absorbance and fluorescence measurements were carried out using a Cytation3 plate reader (BioTek, Winooski, Vermont). The confocal microscopy of nanoparticle-loaded hydrogels used a Nikon Eclipse Ti-A1R microscope (Tokyo, Japan) with an Apo LWD 20×/1.10 water immersion objective, a 561-nm laser, and a 570- to 620-nm filter. The SEM of hydrogels and infected porcine skin was conducted using a Thermo Apreo VS scanning electron microscope (Thermo Fisher Scientific, Hillsboro, Oregon) after preparing samples with critical point drying (Ladd Research Industries, Williston, Vermont). Elastic moduli of hydrogels were assessed by uniaxial compression tests at 25°C using an Instron 5942 universal testing system (Norwood, Massachusetts), with a crosshead speed of 0.1 mm/s and a 500-N load cell. A Rotavapor R-300 (Buchi, Sankt Gallen, Switzerland) was used for preparing dry lipid films, and liposome extrusion was performed using a LiposoFast LF-50 (Avestin, Ottawa, Canada). The hydrodynamic diameter and ζ potential of liposomes were characterized using a Zetasizer Nano ZS90 (Malvern Panalytical, Westborough, MA). Bioluminescence imaging was performed using an IVIS Lumina III imaging system (PerkinElmer, Waltham, Massachusetts) under isoflurane anesthesia.

### Liposome preparation and characterization

HSPC, cholesterol, PE-PEG2000, and PE-LRB were combined in a 200-ml round-bottom flask at a 6.9:2:1:0.1 weight ratio and dissolved in chloroform. The solvent was evaporated at reduced pressure to form a dry lipid film. The film was hydrated in 300 mM (NH_4_)_2_SO_4_ solution containing 150 mM sodium chloride at 65°C to form a multilamellar liposome suspension with a phospholipid concentration of 23 mM. The suspension was sonicated in an ultrasonic bath for 30 min and extruded through a polycarbonate membrane with 100-nm pore size to create unilamellar LIPOs. To establish a transmembrane ammonium gradient, the LIPOs was dialyzed against saline for 24 hours to remove extraliposomal ammonium ions. Ciprofloxacin solution (4 mg/ml in saline) was then mixed with the LIPOs suspension, and the mixture was incubated at 65°C for 30 min for drug loading. The free drug was removed via dialysis for 24 hours yielding cLIPO.

To calculate EE, an aliquot of liposome suspension was diluted 20-fold with 70% (v/v) methanol containing 20 mM HCl to lyse liposomes. The absorbance at 275 nm was measured to determine the amount of drug encapsulated, *D*_encap_, using a standard curve for free ciprofloxacin. EE was determined using Eq. 1, where *D*_t_ is the total mass of drug added

$$\text{EE}\;\left(\%\right)=\left(\frac{D_{\text{encap}}}{D_{t}}\right)\times 100\%$$ (1)

Drug loading (DL%) was also calculated using Eq. 2, where *L* is the dry weight of the liposomes

$$\text{DL}\;\left(\%\right)=\left(\frac{D_{\text{encap}}}{L}\right)\times 100\%$$ (2)

The MIC values of cLIPOs and free ciprofloxacin against *P. aeruginosa* Xen41 and *S. aureus* Xen29 were determined using broth microdilution assays. Drug dilutions were prepared and added to bacterial cultures in microwell plates at a final concentration of 10^5^ CFU/ml in LB medium. Microwell plates were incubated at 37°C with shaking at 100 rpm. After ~20 hours, MICs were determined as the lowest drug concentration at which no visible bacterial growth occurred, determined by measuring absorbance at 600 nm with a plate reader.

### β-Lactamase–cleavable crosslinker synthesis

The β-lactamase–cleavable linker was adapted from previous work with minor modifications (*32*). We briefly describe the synthesis as follows.

#### β-Lactam core (ACLE) modification

ACLE (300 mg, 0.74 mmol) was dissolved in anhydrous DCM (9 ml, 0.14 mmol) and stirred on ice under N_2_. TEA (200 μl, 2.8 mmol) was slowly added to the ACLE mixture over 20 min. NMM (100 μl, 0.0009 mmol) and ATP (150 mg, 0.8 mmol in 1 ml of DCM) were added sequentially thereafter. After stirring for 3 hours, the product was purified using column chromatography with 2% (v/v) methanol in DCM as the eluent to yield the modified β-lactam core.

#### Mal–β-lactam synthesis

We added 3-maleimidepropionic acid (340 mg, 2.01 mmol) and HATU (918 mg, 2.41 mmol) to the previously modified β-lactam core (263 mg, 0.57 mmol) and dissolved in 3 ml of anhydrous DMF. The mixture was stirred for 15 min under N_2_ at 23°C before adding DIPEA (600 μL, 3.52 mmol); the reaction was continued for an additional 75 min. The crude product was partitioned between DCM and water, washed twice with water, and once with brine, then dried over sodium sulfate, filtered, and concentrated by rotary evaporation. The product was purified by column chromatography using 10% (v/v) methanol in DCM as the eluent to obtain maleimide-conjugated β-lactam (mal–β-lactam).

#### Mal–β-lactam deprotection

The *p*-methoxybenzyl protecting group was cleaved to activate the β-lactam ring for β-lactamase recognition. Mal–β-lactam (400 mg, 0.527 mmol) was deprotected in a solution of TFA:anisole:DCM [1:1:5 (v/v/v), 14 ml] under N_2_ on ice for 2 hours. The solvent was then removed under vacuum, and the residue was dissolved in acetone and precipitated in cold diethyl ether, repeated three times. The final product was dried with rotary evaporation.

### Hydrogel preparation and characterization

For R-hydrogel formation, 4-arm-PEG-thiol was dissolved in 1× PBS (pH 6.5). For NR-hydrogels, 4-arm-PEG-thiol was dissolved in 0.1× PBS (pH 3.5) (to maintain gelation times comparable to R-hydrogels). The β-lactamase–cleavable crosslinker solution (for R-hydrogels, 117.2 mg/ml) and mal-PEG-mal crosslinker solution (for NR-hydrogels, 50.5 mg/ml) were prepared in DMSO. For hydrogel labeling, Cy5-maleimide was added to the crosslinker solution at a 1:1000 molar ratio of Cy5 to crosslinker. Hydrogels with a 10% (w/v) polymer density were formed by mixing the crosslinker and PEG at a 1:1 molar ratio of maleimide to thiol, resulting in hydrogels containing 10% (w/w) DMSO in 1× PBS. The mixture was briefly vortexed, and 50 μl was transferred into a 5-mm-diameter and 2-mm-height cylindrical polydimethylsiloxane mold and incubated at 37°C for 1 hour. For liposome-loaded hydrogels, LIPOs or cLIPOs were added to the PEG solution to obtain the desired amount of ciprofloxacin of 7 μg per hydrogel before mixing with the crosslinker. After gelation, hydrogels were thoroughly rinsed with 1× PBS (pH 7.4) to remove DMSO. Each hydrogel was kept in 2 ml of 1× PBS (pH 7.4) for 48 hours for equilibrium swelling before use.

Unreacted thiols in the hydrogel formulations were quantified using a fluorometric thiol quantitation kit, following the manufacturer’s protocols. Crosslinking efficiency was calculated based on the fluorescence values obtained for the hydrogel formulations relative to unreacted 4-arm-PEG-thiol controls. Elastic moduli were determined by compression testing using the linear fitting value of the stress-strain curve within the linear strain range (10 to 15%). Stress was calculated as the force of compression divided by the hydrogel cross-sectional area. The equilibrium swelling ratio (*Q*_s_) was calculated using Eq. 3, where W_w_ is the wet weight of fully swollen hydrogels and *W*_d_ is the weight of lyophilized hydrogels

$$Q_{s}=\left(\frac{W_{w}-W_{d}}{W_{d}}\right)$$ (3)

### Enzymatic degradation of hydrogels

We evaluated the enzymatic degradation of R- and NR-hydrogels in the presence of β-lactamases from *P. aeruginosa* by incubating the hydrogels with enzyme (50 U/ml) in 2 ml of 1× PBS (pH 7.0) at 25°C with shaking at 100 rpm. Digital photographs of the hydrogels were taken at predetermined time points. Concurrently, 100 μl of the incubation solution was collected at each time point to measure the Cy5 and rhodamine B fluorescence. R-hydrogels were similarly tested for potential degradation in the presence of penicillinases from *B. cereus* (10 U/ml), collagenases (10 U/ml), lipases (10 U/ml), hyaluronidases (100 U/ml), phospholipases (12.5 μg/ml), elastases (10 U/ml), and hydrogen peroxide (10 mM).

To assess the bacteria-triggered degradation of R- and NR-hydrogels on nutrient agar, bacteria at a concentration of 10^8^ CFU/ml were streaked onto LB agar. Hydrogels were then placed on the agar and incubated at 37°C until complete degradation of R-hydrogels was observed. Digital photographs of the hydrogels were taken over time. Control hydrogels incubated on nonbacteria inoculated agar were also examined.

### Mammalian cell culture and cytocompatibility studies

NIH 3T3 cells were cultured in DMEM growth media supplemented with 10% (v/v) FBS and 1% (v/v) penicillin/streptomycin and incubated at 37°C with 5% CO_2_. For cytocompatibility studies, NIH 3T3 cells were plated in tissue culture treated 96-well plates at a density of 30,000 cells/cm^2^. After 24-hour incubation, the medium was removed, and cells were exposed to the products of fully degraded hydrogels (R-hydrogel, LIPO-loaded R-hydrogel, and cLIPO-loaded R-hydrogel incubated with β-lactamases from *P. aeruginosa*). Control groups including β-lactamases, cLIPOs, and LIPOs in 1× PBS (pH 7.4), at concentrations equivalent to those found in the degraded hydrogel products, were also included. Cells were then incubated at 37°C with 5% CO_2_ for a further 24 hours. Postincubation, the medium was refreshed, and 10 μl of CCK-8 solution was added to each well. The cells were incubated for 2 hours and absorbance at 450 nm was measured to determine cell viability relative to untreated cells.

For the hemolysis studies, 200 μl of 1% (v/v) RBCs in 1× PBS were incubated with 100 μl of the hydrogel degradation products or controls, as used in the CCK-8 assays, for 1.5 hours at 37°C. RBCs incubated with 2% (v/v) Triton X-100 served as positive controls. After incubation, RBCs were centrifuged for 5 min at 1700*g*, following which 200 μl of the supernatant was transferred to a 96-well plate. Absorbance at 540 nm was measured to determine hemolysis relative to the positive control group.

### Care and use of mice

Four- to 7-week-old male mice (hairless, SKH1) were obtained from the Charles River Laboratories and housed at the Brown University Center for Animal Resources and Education (Providence, RI). All experiments were in accordance with the ethical principles and guidelines approved by the Brown University Institutional Animal Care and Use Committee protocol number 24-05-0001.

### Murine skin abrasion wound infection studies

A mouse model of a skin abrasion wound infection was established using a previously reported technique (*70*, *71*). Bioluminescent *P. aeruginosa* Xen41 was used in these studies. To prepare the bacterial inoculum, bacteria were streaked on LB agar from frozen glycerol stocks, and a single colony was selected for an overnight culture at 37°C and 200 rpm in LB medium. *P. aeruginosa* Xen41 cultures were then refreshed on the morning of infection by a 1:5 dilution into LB and incubated for 3 hours until an optical density at 600 nm of 0.4 was reached (mid-logarithmic phase). The bacteria culture was centrifuged for 10 min at 3000*g* at room temperature (21°C) twice, and the pellet was resuspended in 300 μl of saline to achieve a concentration of ~10^9^ CFU/ml.

Mice were anesthetized with 2% (v/v) isoflurane. Under aseptic conditions, a superficial wound ~1 cm by 1 cm was created on the mouse dorsal midline by stripping the epidermal layer nine times with Tensoplast adhesive tape (1 cm by 1 cm), resulting in visible skin reddening and glistening, but no bleeding. The wound was inoculated with 10 μl of bacteria and immediately covered with a medical-grade transparent film dressing (Tagderm, 3M). After 2 hours, the transparent film was partially removed, and the hydrogels were placed on the wound. The mouse weight and temperature were monitored daily. Xen41 bioluminescence and fluorescence signals for Cy5 and LRB were assessed 2 hours posttreatment on day 0 and then daily for 4 days using an IVIS system. On day 4 posttreatment, the dorsal skin was harvested and homogenized in 1 ml of sterile saline using a bead beater. The skin homogenate was serially diluted and plated on LB agar for CFU enumeration. Skin samples were also collected for histological analysis. The tissues were fixed in 4% (v/v) paraformaldehyde and dehydrated in 75% (v/v) ethanol before H&E staining and macrophage immunostaining, which were performed at the Molecular Pathology Facility at Brown University.

### Ex vivo porcine burn wound infection studies

Porcine skin tissue was obtained from the backs of euthanized Göttingen minipigs provided by the Brown University Center for Animal Resources and Education (Providence, RI) and stored at −20°C. After thawing at 21°C, the skin was sterilized with 70% (v/v) ethanol for 30 min. Burn wounds were created with a heated copper rod (8-mm diameter), applied to the skin for 30 s. Ex vivo wounds were inoculated with Xen41 or Xen29 (10^7^ CFU) and incubated at 37°C for 24 hours. cLIPO-loaded R-hydrogels were then applied to the wounds and further incubated at 37°C for 24 hours. Daily digital images were taken to evaluate hydrogel degradation. Controls without treatment were included for bacterial burden comparison. For SEM imaging, samples were fixed in PBS with 2% (v/v) formaldehyde and 2% (v/v) glutaraldehyde, dehydrated through a graded ethanol series [50, 75, 90, and 100% (v/v)], and dried using a critical point dryer.

### Evaluation of drug-resistance development

The development of drug resistance was investigated on ex vivo burn wounded porcine skin infected with 10^7^ CFU of Xen29 for 12 hours at 37°C. For each cycle of the study, cLIPO-loaded R-hydrogels were applied to the wound and incubated at 37°C. After 12 hours, bacteria were collected from the skin by gently rubbing with a cotton swab and inoculating on LB agar plates, which were incubated for 12 hours. The colonies that grew were then cultured in 5 ml of LB and used to inoculate skin burn wounds for the next cycle of the study. After each cycle, the ciprofloxacin MIC of the cultured bacteria was also evaluated using a microdilution assay to evaluate any change in MIC. For comparison, Xen29 was also exposed to a sub-MIC concentration of ciprofloxacin (i.e., 0.1 μg/ml) in 5 ml of LB media and incubated for 12 hours. Following this, 50 μl of the bacterial culture was transferred to fresh media containing ciprofloxacin for a total of 10 cycles. After each cycle, the bacteria were streaked onto LB agar for subsequent MIC determination.

### Statistical analysis

All experiments were performed in triplicate or more. Data are presented as average ± SD. All statistical analyses were performed with GraphPad Prism 10.1 software using unpaired two-tailed Student’s *t* test and one- or two-way analysis of variance (ANOVA) with Tukey’s post hoc analysis (α = 0.05; *P < 0.05, **P < 0.01, ***P < 0.001, and ****P < 0.0001; matching letters indicate *P* < 0.05).
