# Supplementary material for: Bacterial enzyme-responsive hydrogels for triggered delivery of antibiotics to infected wounds
Source: Sci Adv. 2026 Mar 20;12(12):eadz0786. doi: 10.1126/sciadv.adz0786 (PMC13004048; doi:10.1126/sciadv.adz0786)
Supplement: Supplementary file 1 — Figs. S1 to S13 Table S1 [file sciadv.adz0786_sm.pdf]

Science Advances
AAAS

Supplementary Materials for

Bacterial enzyme-responsive hydrogels for triggered delivery of antibiotics to infected wounds

Akram Abbasi *et al.*

Corresponding author: Anita Shukla, anita_shukla@brown.edu

Sci. Adv. **12**, eadz0786 (2026)
DOI: 10.1126/sciadv.adz0786

This PDF file includes:

Figs. S1 to S13
Table S1

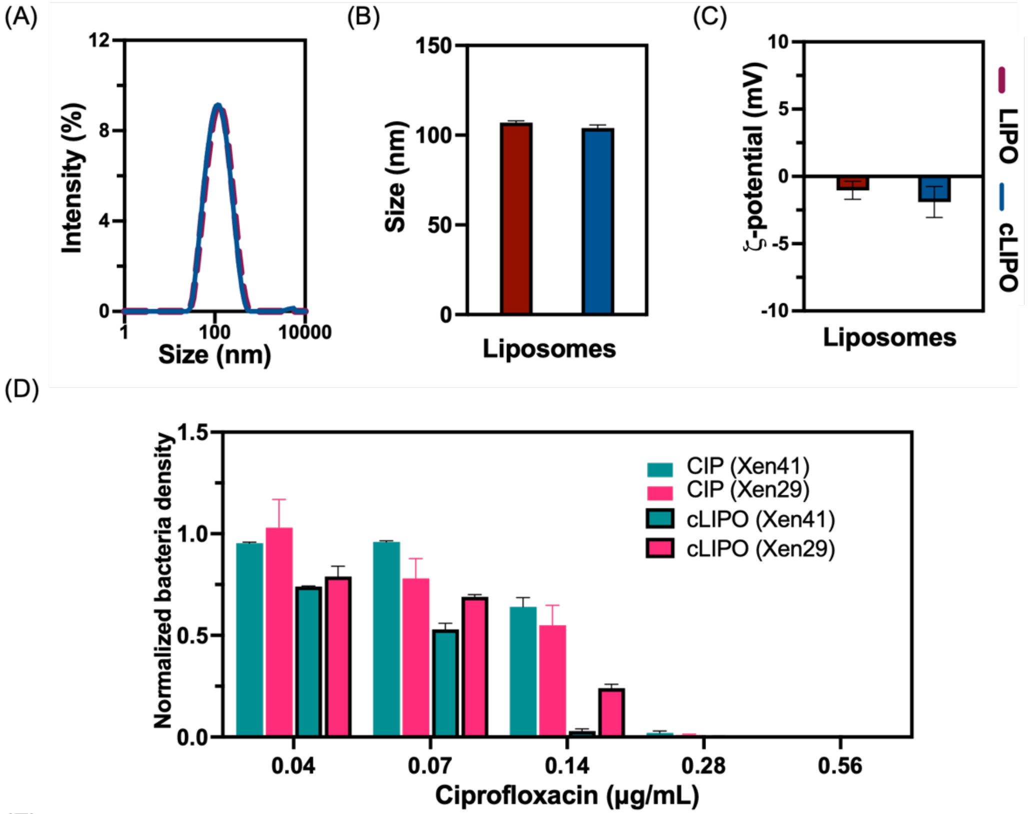

(E)

Liposome	Size (nm)	PDI	ζ-potential (mV)	EE %	DL % (w/w)	MIC (μg/mL)
**LIPO**	107.2 ± 1.0	0.23 ± 0.01	-1.0 ± 0.67	N.A.	N.A.	N.O.
**cLIPO**	104.1 ± 1.7	0.23 ± 0.00	-1.9 ± 1.15	87.6 ± 5.3	7.5 ± 0.3	0.3

Fig. S1. Characterization of liposomes. (**A**) Hydrodynamic diameter distribution of liposomes as determined by DLS. (**B**) Average hydrodynamic diameter and **(C)** ζ-potential of liposomes in 1× PBS (pH 7.4). Data are presented as average ± standard deviation (n = 3). Statistical analysis was performed using an unpaired two-tailed Student's t-test ($p > .05$, not significant). (**D**) Normalized bacteria density of *P. aeruginosa* Xen41 and *S. aureus* Xen29 treated with free ciprofloxacin (CIP) or cLIPO. (**E**) Summary table of liposome characterization. Data is presented as average ± standard deviation (n = 3).

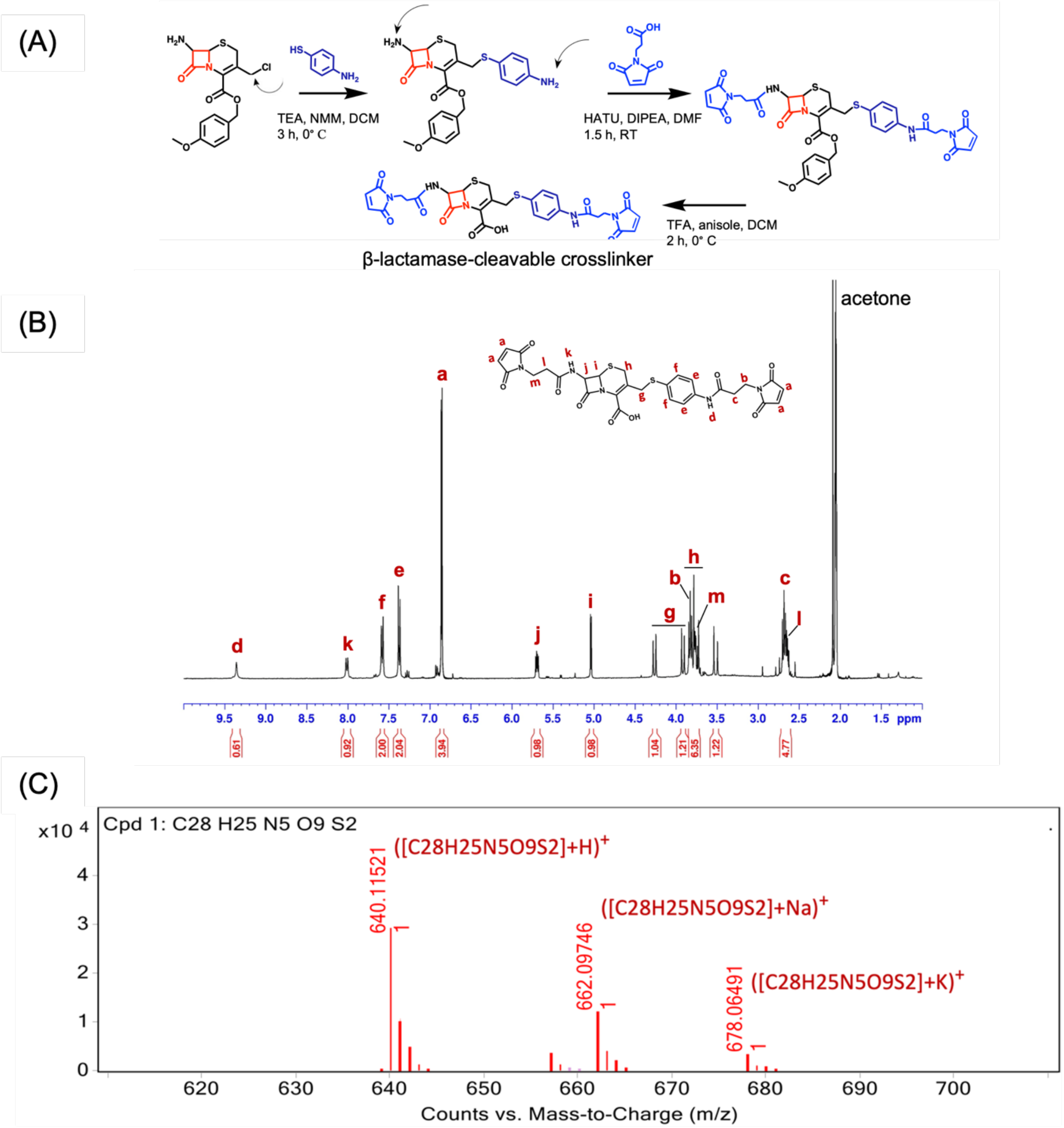

Fig. S2. Synthesis and characterization of the β-lactamase-cleavable hydrogel crosslinker. (**A**) Schematic illustration of β-lactamase-cleavable crosslinker synthesis. (**B**) ^{1}H-NMR of β-lactamase-cleavable crosslinker acquired using a Bruker DRX Avance 400 MHz spectrometer. ^{1}H-NMR (400 MH, acetone-d_6) δ(ppm): 9.34 (s, 1H), 8.02 (d, J = 8.5 Hz, 1H), 7.57 (d, J = 8.6 Hz, 2H), 7.37 (d, J = 8.6 Hz, 2H), 6.85 (d, J = 5 Hz, 4H), 5.71 - 5.66 (dd, J_1 = 4.7Hz, J_2 = 9 Hz, 1H), 5.03 (d, J = 4.8 Hz, 1H), 4.28 - 3.89 (dd, J_1 = 13.3 Hz, J_2 = 136.6 Hz, 2H), 3.84 - 3.69 (m, 5H), 3.51 (d, J = 17.7 Hz, 1H), 2.70-2.61 (m, 4H). (**C**) High resolution mass spectrometry (HRMS) of β-lactamase-cleavable crosslinker. HRMS m/z calculated for $C_{28}H_{25}N_5O_9S_2$ [M+H]: 640.1170; found 640.1152.

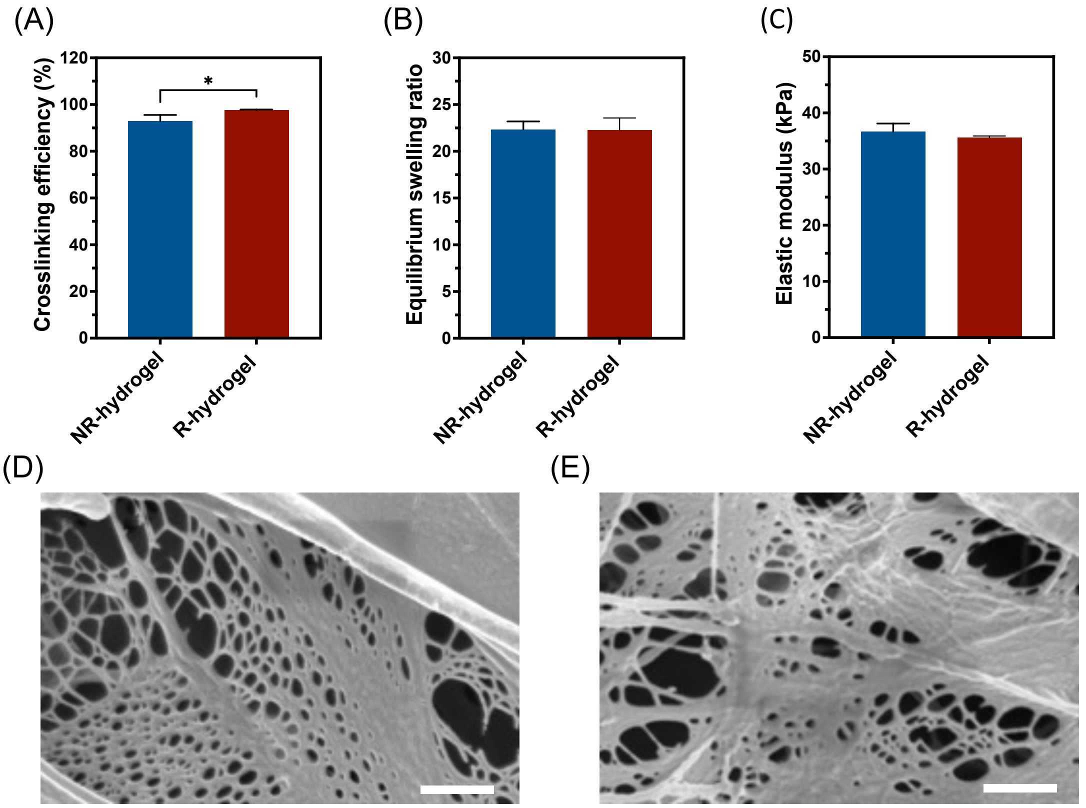

Fig. S3. Physical characteristics of hydrogels. (**A**) Crosslinking efficiency, (**B**) equilibrium swelling ratio, and **(C)** elastic modulus of NR- and R-hydrogels. Representative SEM images of the (**D**) NR-hydrogel and (**E**) R-hydrogel. Scale bars = 500 nm. Data is presented as average ± standard deviation (n = 3). Statistical analysis was performed using an unpaired two-tailed t-test (**p* < 0.05).

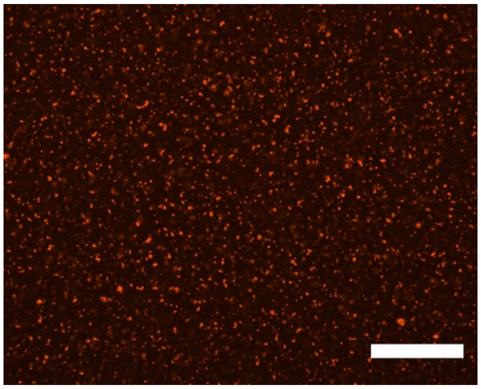

Fig. S4. Liposome distribution within R-hydrogel. Representative fluorescence microscopy image of cLIPO distributed inside cLIPO-loaded R-hydrogel (red = LRB). Scale bar = 100 µm.

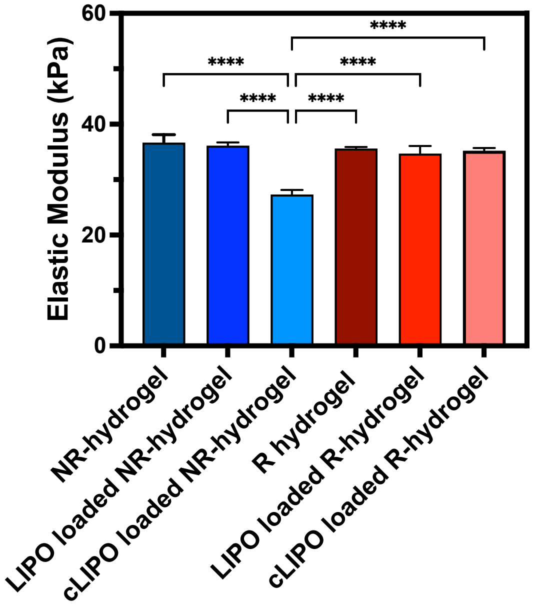

Fig. S5. Elastic modulus of different hydrogel formulations. Data is presented as average ± standard deviation (n = 3). Statistical analysis was performed using one-way ANOVA with Tukey's post-hoc analysis (****$p < 0.0001$).

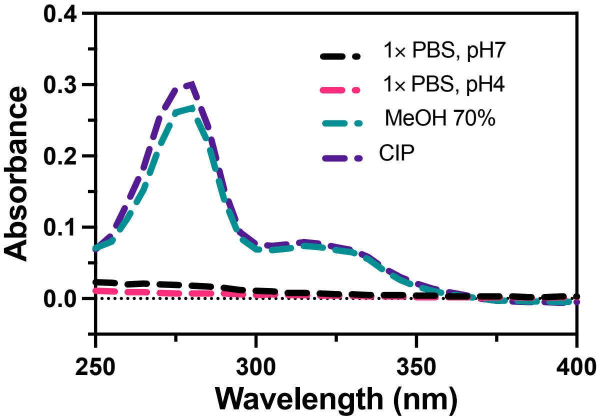

Fig. S6. Stability of cLIPO within cLIPO-loaded R-hydrogels. cLIPO-loaded R-hydrogels were incubated in 1× PBS at pH 7 and pH 4 for 7 days. Ciprofloxacin (CIP) release was assessed by measuring absorbance spectra of the incubation media, with CIP absorbance detected at 275 nm as a measure of liposome disruption. Significant CIP release was only observed in the presence of 70% methanol, indicating liposome rupture and subsequent CIP release under this condition.

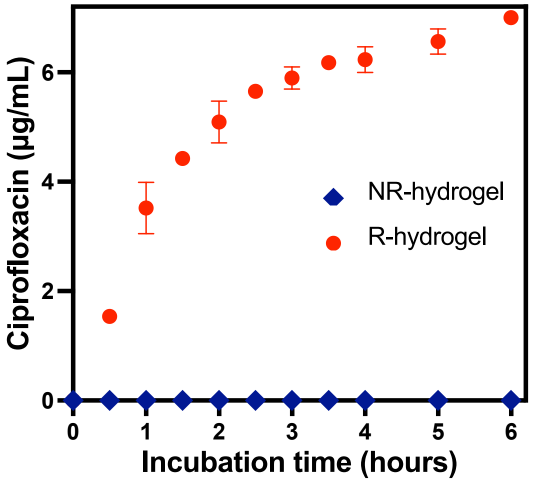

Fig. S7. Theoretical ciprofloxacin release over time from R- and NR-hydrogels. Estimated ciprofloxacin release from R-hydrogels upon exposure to β-lactamases (50 U/mL cephalosporinases from *P. aeruginosa*) was determined based on the ciprofloxacin loading of cLIPO within R-hydrogels (7 μg) and the corresponding normalized LRB fluorescence intensity from the LIPO release profile. Data are presented as average ± standard deviation (n > 3).

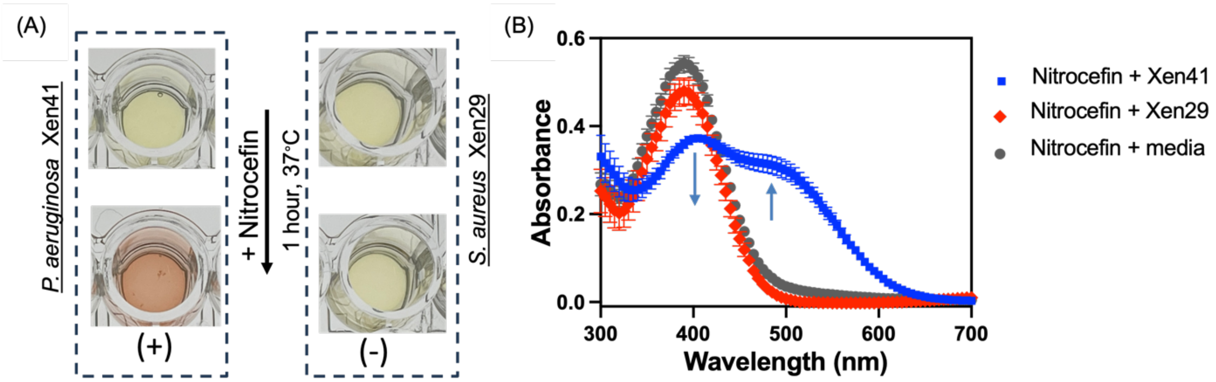

Fig. S8. Detecting β-lactamase production by *P. aeruginosa* Xen41 and *S. aureus* Xen29. (**A**) Bacteria incubation with nitrocefin results in a color change from yellow to red upon hydrolysis of the chromogenic nitrocefin substrate by β-lactamases. This color shift was observed only with *P. aeruginosa* Xen41. (**B**) Absorbance spectra analysis confirmed nitrocefin hydrolysis by Xen41, evidenced by a decrease in the characteristic 390 nm peak and the emergence of a new peak at 490 nm. No such spectral change was observed with *S. aureus* Xen29 or media (no bacteria), indicating a lack of β-lactamase activity in those conditions.

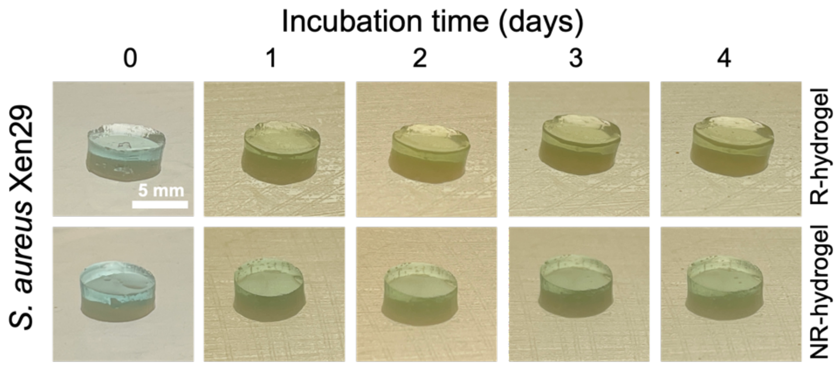

Fig. S9. β-lactamase-induced degradation of R-hydrogels *in vitro*. R-hydrogels exhibited no degradation response when incubated on agar inoculated with *S. aureus* Xen29, a non-β-lactamase-producing bacterial strain.

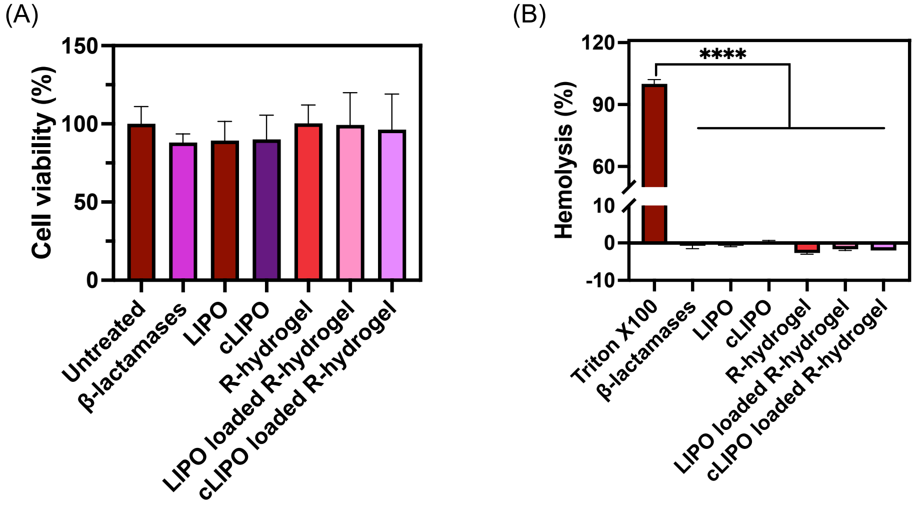

Fig. S10. Cytocompatibility of R-hydrogel degradation products. (**A**) Cell viability of NIH 3T3 fibroblasts and (**B**) RBC hemolysis after incubation with the degradation products of R-hydrogel formulations exposed to β-lactamases from *P. aeruginosa* in 1× PBS (pH 7.4). Data is presented as average ± standard deviation (n=4). Statistical analysis was performed using one-way ANOVA with Tukey's post-hoc analysis (****$p < 0.0001$).

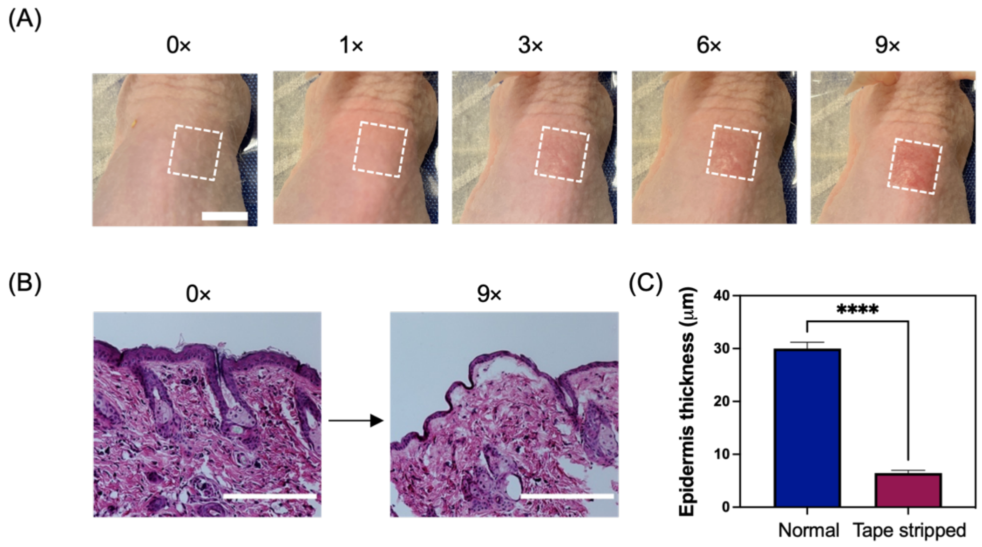

Fig. S11. Establishing murine skin abrasion wound model. (**A**) Repeated tape stripping (up to nine times) resulted in visible reddening and glistening of the skin and no bleeding, establishing the wound. Scale bar = 1 cm. (**B**) Representative histology images of H&E-stained murine skin before (0×) and after (9× tape strips) wound formation, showing reduced epidermal thickness following tape stripping. Scale bars = 200 µm. (**C**) An average reduction of 25 µm in the epidermal thickness was observed following nine tape strips. Data is presented as average ± standard deviation (n=3). Statistical analysis was performed using an unpaired two-tailed t-test (****$p <$ 0.0001).

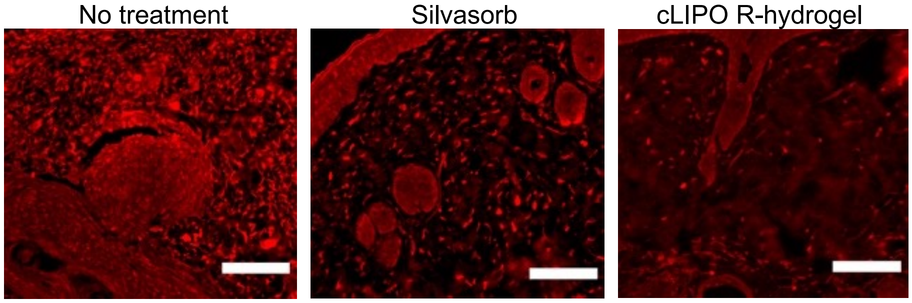

Fig. S12. Immunofluorescence staining of the harvested murine skin tissue at 4 days post-infection and treatment. Representative images of harvested skin tissues stained for macrophages (red). Scale bars = 100 µm.

Table S1. Overview of blinded histology scoring system.

Physiological parameter	Stage	Score
**Re-epithelialization**	Complete	2
	Partial	1
	None	0
**Epidermal thickness**	Normal	2
	Hypertrophy	1
	Hypoplasia	0
**Keratinization**	Yes	2
	No	0
**Granulation tissue (GT)**	Intact dermis	2
	Thick GT	1
	Thin GT	0
**Remodeling/ Normal pathology**	Complete	2
	Partial	1
	None	0
**Inflammation**	None	2
	Some	1
	Abundant	0

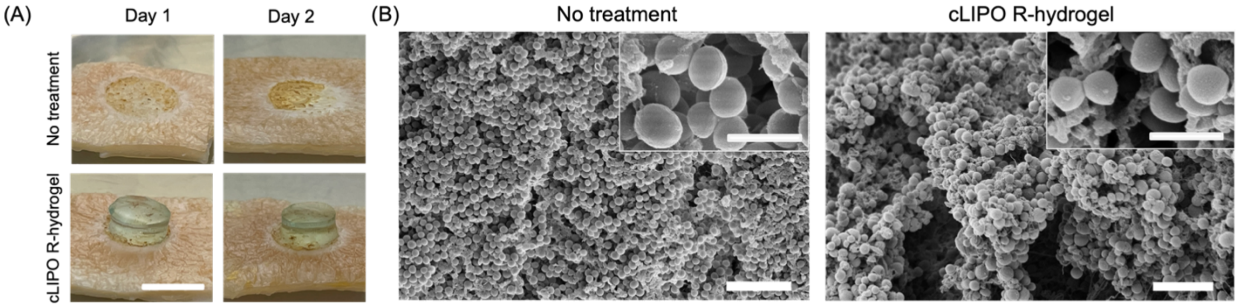

Fig. S13. cLIPO-loaded R hydrogels on *ex vivo S. aureus* Xen29 infected porcine burn wound infection model. (**A**) Representative digital photographs of cLIPO-loaded R-hydrogels applied to *ex vivo S. aureus* Xen29 infected burn wounds, showing no visible hydrogel degradation. The no-treatment group served as the control. Images are representative of four independent experiments. Scale bar = 8 mm. (**B**) SEM images on day 1 revealed a dense *S. aureus* Xen29 biofilm on both untreated and hydrogel-treated infected skin. Scale bars = 5 µm. Insets show the bacterial morphologies; scale bars = 1 µm.
